# Genome Sequence of Vibrio campbellii Strain UMTGB204, a Marine Bacterium Isolated from a Green Barrel Tunicate

**DOI:** 10.1128/genomeA.00210-15

**Published:** 2015-03-26

**Authors:** Huan You Gan, Mohd Ezhar Mohd Noor, Nur Azna Saari, Najiah Musa, Baharim Mustapha, Gires Usup, Muhd Danish-Daniel

**Affiliations:** a Genomics Facility, Monash University Malaysia, Bandar Sunway, Petaling Jaya, Selangor, Malaysia; b School of Science, Monash University Malaysia, Bandar Sunway, Petaling Jaya, Selangor, Malaysia; c School of Fisheries and Aquaculture Sciences, Universiti Malaysia Terengganu, Kuala Terengganu, Terengganu, Malaysia; d Institute of Tropical Aquaculture, Universiti Malaysia Terengganu, Kuala Terengganu, Terengganu, Malaysia; e Faculty of Science and Technology, School of Environmental Science and Natural Resources, Universiti Kebangsaan Malaysia, Bangi, Selangor, Malaysia

## Abstract

Vibrio campbellii strain UMTGB204 was isolated from a green barrel tunicate. The genome of this strain comprises 5,652,224 bp with 5,014 open reading frames, 9 rRNAs, and 116 tRNAs. It contains genes related to virulence and environmental tolerance. Gene clusters for the biosynthesis of nonribosomal peptides and bacteriocin were also identified.

## GENOME ANNOUNCEMENT

Vibrio campbelli is widely distributed in marine environments (1). This gammaproteobacteria is an important pathogen to many aquatic organisms from both wild and cultured systems, most notably peneid shrimp, several fish species, and mollusks (2, 3). Vibrio campbelli strain UMTGB204 was isolated from a green barrel tunicate in Bidong Island, a coral reef island in the South China Sea. The genome of this strain was sequenced in order to gain insights into its relationships with tunicates.

V. campbellii strain UMTGB204 was cultured in Marine Broth 2216 (Difco). Genomic DNA was then extracted using the GF-1 nucleic acid extraction kit (Vivantis, Malaysia). Sequencing was performed on the Illumina HiSeq2000 platform, generating 33,271,099 raw FASTQ paired-end reads. Two million reads were subsampled for error correction and *de novo* assembled using SPAdes version 3.1.0 (4). The resulting contigs were used for scaffolding and then gap-closed using SSPACE version 2.0 and GapFiller version 1.11 (5, 6). Sixty gap-filled contigs >3 kb with an *N*_50_ of 947,033 bp were produced, and the total sequence length was 5,652,224 bp with 70× coverage.

The Prokka version 1.8 annotation pipeline, comprising Prodigal version 2.60, RNAmmer version 1.2, and Aragorn version 1.2.36, was used to annotate the genome, predicting 5,014 open reading frames, 9 rRNAs, and 116 tRNAs (7–10). The predicted 16S rRNA was queried with BLASTn (11) against the nucleotide collection database, confirming that the strain was Vibrio campbellli. Further validation of the species was performed using *in silico* genome-to-genome comparison of the UMTGB204 strain to Vibrio campbellli 200612B (GenBank accession no. BANY00000000.1), showing a DNA-DNA hybridization probability of 90.03% (12). InterProScan5 was used to provide additional annotation to the predicted protein sequences (13). Furthermore, antiSMASH was used to identify the presence of secondary metabolite biosynthesis gene clusters in the genome (14).

Similar to the genomes of other Vibrio campbelli strains, strain UMTGB204 carries genes that are responsible for virulence—such as the hemolysin, ToxR, and secretion systems (15, 16)—but unlike genomes of other reported strains, the proteorhodopsin-related gene was not identified in the genome of strain UMTGB204. Analysis of the genome also revealed genes associated with environmental adaptation, including osmotic and oxidative stress, thermal shock, and siderophores. Interestingly, several gene clusters for the biosynthesis of nonribosomal peptides and bacteriocin have been identified (contigs 1, 2, 10, and 19). This suggests that, as a survival mechanism in the marine environment, strain UMTGB204 possesses a better ability to compete with other closely related vibrios to colonize the host tunicate.

### Nucleotide sequence accession number.

This whole-genome shotgun project has been deposited in DDBJ/EMBL/GenBank under the accession number JSFE00000000.
